# *SYNJ1* gene associated with neonatal onset of neurodegenerative disorder and intractable seizure

**DOI:** 10.1002/mgg3.341

**Published:** 2017-11-27

**Authors:** Nuha Al Zaabi, Noora Al Menhali, Fatma Al‐Jasmi

**Affiliations:** ^1^ Department of Pediatric College of Medicine and Health Science United Arab Emirates University Al Ain UAE; ^2^ Department of Pediatric Mafraq Hospital Abu Dhabi UAE

**Keywords:** intractable seizure, neurodegenerative disorder, *SYNJ1*, whole‐genome sequencing

## Abstract

**Background:**

Synaptojanin 1 is encoded by the *SYNJ1*(MIM 604297) and plays a major role in phosphorylation and recycling of synaptic vesicles. Mutation of *SYNJ1* is associated with two distinct phenotypes; a known homozygous missense mutation (p.Arg258Gln) associated with early‐onset Parkinson disease (MIM 615530), whereas mutation with complete loss of *SYNJ1* function result in a lethal neurodegenerative disease with intractable seizure and tauopathies (MIM 617389).

**Methods:**

We report two related children from consanguineous family presented with intractable seizure, profound developmental delay, failure to thrive, acquired microcephaly, and hypotonia. The brain MRI is normal and EEG showed hypsarrhythmia.

**Result:**

The diagnosis was achieved via whole‐genome sequencing which showed homozygous mutation in *SYNJ1* (c.709C>T, p.Gln237*).

**Conclusion:**

A clinical pattern of neonatal‐onset intractable seizure, profound developmental delay, muscular hypotonia, hypsarrhythmia, and no focal abnormality of brain MRI should prompt initiation of molecular genetic analysis of *SYNJ1*. Establishment of the diagnosis permits genetic counseling, prevents patients undergoing unhelpful diagnostic procedures and allows for accurate prognosis.

## 1 INTRODUCTION

Synaptojanin is a polyphosphoinositide phosphatase, which presents in presynaptic nerve terminals and compounds to proteins involved in endocytosis. It has a major role in phosphorylation and recycling of synaptic vesicles (Harris, Hartwieg, Horvitz, & Jorgensen, 2000). This function is facilitated by two consecutive phosphatase domains in the Synaptojanin: an N‐terminal Sac1‐like inositol domain (Sac1) and a central 5′‐phosphatase domain (5′PP), which enable the removal of a phosphate group from the 4 and 5 position of phosphatidylinositol 4,5‐bisphosphate [PI (4,5) P2] (Di Paolo & De Camilli, 2006; McPherson, Garcia, Slepnev, & David, 1996).

Synaptojanin 1 is encoded by the *SYNJ1* on chromosome 21q22.11 and it is highly conserved throughout evolution (Cremona et al., 2000). Mutation in the *SYNJ1* is associated with two different rare neurological diseases; early‐onset Parkinson disease (MIM 615530) and severe neurodegenerative with intractable seizure and tauopathies (MIM 617389) (Drouet & Lesage, 2014; Dyment et al., 2015).

Early‐onset Parkinson disease was described in three independent families with same homozygous missense variant (R258Q) resulting in loss of dephosphorylation activity of Sac1 domain (Krebs et al., 2013; Olgiati et al., 2014). Another homozygous mutation R459P in *SYNJ1* was identified in an Indian family (Kirola, Behari, Shishir, & Thelma, 2016). Recently, Taghavi et al., (2017) published a *SYNJ1* p.R839C mutation in two Iranian siblings with poorly levodopa‐responsive Parkinsonism and generalized seizures since 24 years of age associated with longitudinal tongue fissures. All affected individuals showed early progressive Parkinsonism symptoms in their twenties in combination with cognitive decline or early‐onset refractory seizures (Krebs et al., 2013; Olgiati et al., 2014).

Recently, new devastating phenotype was reported as result of complete loss of *SYNJ1* function or formation (Neurodegenerative disease with intractable seizure and tauopathies) (Dyment et al., 2015; Hardies et al., 2016).

Here, we report two related patients from consanguineous Emirati family of Omani origin presenting with neonatal onset of intractable seizures and a neurodegenerative disease course. Whole‐genome sequencing revealed novel homozygous nonsense mutation in the *SYNJ1*. These findings are consistent with what has been previously described in patients with *SYNJ1* and expand our knowledge of this newly identified disease.

## 2 CASE PRESENTATION

**Figure 1:**
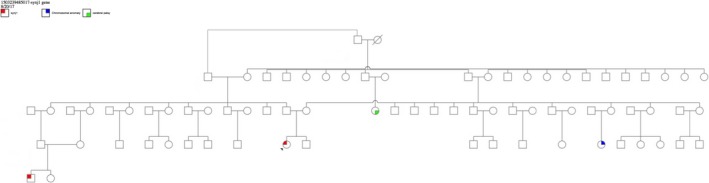
Family pedigrees case 1. The arrow point to the proband case who underwent whole‐genome sequencing

Case 1: Proband is a 2‐year‐old girl who was born term to first cousin parent after an uneventful pregnancy (Figure 1). Patient had normal head circumference. In the second day of life she had seizure in the form of lip smacking for few seconds followed by multiple episodes of tonic movements of upper and right lower extremities with head deviation to one side lasted for 1 to 2 minutes. EEG showed hypsarrythmia pattern, a trial of prednisolone for 1 month resulted in partial control of seizure for short period. Multiple antiepileptic medications were tried with poor control, including phenobarbitone, sodium valporoate, levertiracim, clobazam, vigabatrin, and topiramate.

The proband has severe global developmental delay. She did not gain any skills; there is no head support, no social smile, not fixing or following and cannot sit or stand. She is a calm child and not difficult to handle.

Parent denied any history of swallowing or feeding difficulties. Physical examination of the 2‐year‐old patient showed acquired microcephaly and failure to thrive (head circumference: 43 cm, weight: 7 kg which are below 3rd centile) bedridden and not interactive or interested on the surroundings, no dysmorphic features. She has scoliosis. There is significant truncal and peripheral hypotonia, and persistent palmer and planter reflexes.

The microarray showed deletion of chromosome 19q13.12, but the healthy father had the same finding. Brain MRI showed no evidence of ischemia or brain anomalies. The result of whole‐exome sequence was not conclusive. The whole‐genome sequencing at Centogene identified homozygous mutation in *SYNJ1* (NM_003895.3) (c.709C>T, p.Gln237*).

Case 2: This is a 2‐year‐old boy (cousin of case 1) with global developmental delay. On neurological examination, patient noted to have acquired microcephaly (head circumference, 45.3 cm (20th centile) at 1 year, and 46.5 cm (7th centile) at 2 year; dropped two centiles on head growth chart), not dysmorphic, with no visual fixation or tracking. There is drooling and witnessed nasal regurgitation indicative of dysphagia and palatal insufficiency, respectively. Motor exam showed marked head lag and axial hypotonia with hyperreflexia and clonus. There are no cutaneous features of spinal dysraphism or neurocutaneous stigmata.

EEG showed hypsarrhythmia and epileptic spasms; features consistent with West syndrome. Patient developed other type of seizures including generalized tonic clonic seizures and myoclonus. All seizure types are pharmacoresistant. Brain MRI showed no focal brain abnormality with mild nonspecific widening of the ventricles and subarachnoid spaces. Targeted mutation analysis for *SYNJ1* (NM_003895.3) showed the same familial homozygous mutation.

## 3 DISCUSSION AND CONCLUSIONS

This report describes the genotype and phenotype of two related patients from consanguineous family with mutation in *SYNJ1* identified by whole‐genome sequencing. These patients presented with neonatal‐onset intractable seizure, profound developmental delay, feeding difficulties, severe failure to thrive, acquired microcephaly, and hypotonia. The most striking pathological changes in this condition are the deposition of highly phosphorylated neurofibrillary tangles situated in various brain tissues, predominantly in substantia nigra (Hardies et al., 2016). The homozygous (c.709C>T, p.Gln237*) mutation identified in our patients is localized in the Sac 1 domain and is expected to result in loss of protein formation. A similar case was previously reported with premature stop variant p.Arg136* identified in a Pakistani patient of consanguineous parents who manifested with early‐onset refractory epilepsy, characterized by hypsarrythmia. A magnetic resonance imaging conducted at age of 5 years revealed no structural brain malformation with mild cerebral atrophy. He had showed profound psychomotor decline and died at age of 6.5 year. The autopsy reveled significant findings in the substantia nigra. There were neuronal loss and neurofibrillary degeneration with positive immunohistochemistry for tau protein (Table 1) (Dyment et al., 2015). Another study reported three families with early onset refractory seizures, progressive neurological decline and early mortality (Hardies et al., 2016). Therefore, there are total of seven patients from four different families reported with this severe phenotype. All those patients are from middle east except family 4 who are Caucasian nonconsagnuinous with compound heterozygous. The *SYNJ1* mutations associated with this devastating phenotype are nonsense, two premature stop variants in a compound heterozygous state in addition to a homozygous missense variant (p.Tyr888Cys, Family 2) located in the 5′PP domain leading to a decreased dephosphorylation capacity toward all substrates. These patients presented in the neonatal period with hypotonia, intractable seizure, and severe developmental delay in addition to feeding difficulties. The life spans of these patients are short and died in the first decade. Some of those patients had skeletal manifestation such as scoliosis and multiple contracture. All the patients had initially normal brain MRI and the EEG showed modified hypsarrhythmia (Table 1). This clinical presentation should prompt initiation for mutation analysis of *SYNJ1*, which will facilitate early diagnosis and prevention of this disease in the family.

**Table 1:** Summary of patients' clinical presentations and comparison to the published patients

	Patient 1	Patient 2	Family 1^a^	Family 2^b^		Family 3^b^		Family 4^b^	
Ethnicity and consanguinity	Oman, first cousin	Oman, consanguineous	Pakistan, first cousin	Moroccan, consanguineous		Moroccan, consanguineous		Caucasian, nonconsanguineous	
Mutation	c.709C>T, p.Gln237*	c.709C>T, p.Gln237*	c.406C>T, p.Arg136*	c.2663A>G, p.Tyr888Cys		c.2528G>A, p.Trp843*		c.1938delT/c.3365‐2A>G p.Gln647Argfs*6/p.Ser1122Thrfs*6	
Gender	F	M	M	F	M	F	F	M	M
Age at onset	2 days	NA	9 days	3 days	2 days	1st day	1st day	12 day	1st day
Age at examination	2 years	2 years	NA	7 years	6 years	5 years	2.5 year	Died at 2.5 year	Died at 8 year
Feeding issue	None so far	Yes	Yes	Yes	Yes	Yes	Yes	Yes	Yes
Developmental delay	Yes	Yes	Yes	Yes	Yes	Yes	Yes	Yes	Yes
Nonverbal	Yes	Yes	Yes	Yes	Yes	Yes	Yes	NA	Yes
Intractable Seizure	Yes	Yes	Yes	Yes	Yes	Yes	Yes	Yes	Yes
Hypotonia	Yes	Yes	Yes	Yes	NA	Yes	Yes	Yes	No
EEG	Hypsarrhythmia	Hypsarrhythmia	Modified hypsarrhythmia	Modified hypsarrhythmia or multifocal epileptic activity on a slow background	Multifocal epileptic activity on a slow background	Modified hypsarrhythmia	Focal spikes on a slow background	Multifocal spike discharges and abnormal background	Multifocal epileptic activity on a slow background
Brain imaging	Normal	Mild cerebral atrophy	Mild cerebral atrophy	Normal	Normal	Normal	Normal	Normal	Thin corpus callosum and limited gliosis and atrophy of the periventricular white matter in the youngest brother
Other findings	Scoliosis		ETC. studies: low complex I, brain biopsy showed tau pathology to substantia nigra. Multiple contractures	Progressive spastic quadriplegia, central visual impairment		Increased CK and lactate. Combined deficiency of complex I and II activities in muscle biopsy		Scoliosis	

M, male; F, female; ETC, electron transport chain; NA, not available.

a Dyment et al. (2015).

b Hardies et al. (2016).

Synaptojanin 1 is a phosphoinositide phosphatase protein, which functions to facilitate the proper synaptic interaction. *SYNJ1* was discovered to be a causative gene in early‐onset atypical Parkinsonism and also in neonatal‐onset refractory seizure and neurodegenerative disease. Synaptojanin 1 serves to organize synaptic vesicle recycling and endocytosis; thus understanding of these pathways is of significant importance to explore the neurodegenerative process evolved and to find potential therapeutic agents.

## ETHICAL COMPLIANCE

Signed informed consent was obtained from the patient's parents in accordance with the Al Ain Medical District Human Research Ethics Committee (AAMDHREC). This study was exempted from Ethical approval.

## CONFLICT OF INTEREST

Authors declare no conflict of interest.

## References

[mgg3341-bib-0001] Cremona, O. , Nimmakayalu, M. , Haffner, C. , Bray‐Ward, P. , Ward, D. C. , & De Camilli, P. (2000). Assignment of SYNJ1 to human chromosome 21q22.2 and Synj12 to the murine homologous region on chromosome 16C3‐4 by in situ hybridization. Cytogenetics and Cell Genetics, 88(1–2), 89–90. 10773674 10.1159/000015493

[mgg3341-bib-0002] Di Paolo, G. , & De Camilli, P. (2006). Phosphoinositides in cell regulation and membrane dynamics. Nature, 443, 651–657. 17035995 10.1038/nature05185

[mgg3341-bib-0003] Drouet, V. , & Lesage, S. (2014). Synaptojanin 1 mutation in Parkinson's disease brings further insight into the neuropathological mechanisms. BioMed Research International, 2014, 1–9. 10.1155/2014/289728 PMC4181773 25302295

[mgg3341-bib-0004] Dyment, D. A. , Smith, A. C. , Humphreys, P. , Schwartzentruber, J. , Beaulieu, C. L. , Bulman, D. E. , … FORGE Canada Consortium . (2015). Homozygous nonsense mutation in SYNJ1 associated with intractable epilepsy and tau pathology. Neurobiology of Aging, 36(2), 1222. e1‐5. 10.1016/j.neurobiolaging.2014.09.005 25316601

[mgg3341-bib-0005] Hardies, K. , Cai, Y. , Jardel, C. , Jansen, A. C. , Cao, M. , May, P. , … Bhambhani, V. (2016). Loss of SYNJ1 dual phosphatase activity leads to early onset refractory seizures and progressive neurological decline. Brain, 139(Pt 9), 2420–2430. 27435091 10.1093/brain/aww180 PMC4995362

[mgg3341-bib-0006] Harris, T. W. , Hartwieg, E. , Horvitz, H. R. , & Jorgensen, E. M. (2000). Mutations in synaptojanin disrupt synaptic vesicle recycling. Journal of Cell Biology, 150, 589–599. 10931870 10.1083/jcb.150.3.589 PMC2175188

[mgg3341-bib-0007] Kirola, L. , Behari, M. , Shishir, C. , & Thelma, B. K. (2016). Identification of a novel homozygous mutation arg459pro in SYNJ1 gene of an Indian family with autosomal recessive juvenile parkinsonism. Parkinsonism and Related Disorders, 31, 124–128. 27496670 10.1016/j.parkreldis.2016.07.014

[mgg3341-bib-0008] Krebs, C. E. , Karkheiran, S. , Powell, G. C. , Cao, M. , Makarov, V. , Darvish, H. ,… De Camilli, P. (2013). The sac1 domain of SYNJ1 identified mutated in a family with early‐onset progressive parkinsonism with generalized seizures. Human Mutation, 34(9), 1200–1207. 23804563 10.1002/humu.22372 PMC3790461

[mgg3341-bib-0009] McPherson, P. S. , Garcia, E. P. , Slepnev, V. I. , & David, C. (1996). A presynaptic inositol‐5′‐phosphatase. Nature Reviews Neuroscience, 379, 353–357. 10.1038/379353a0 8552192

[mgg3341-bib-0010] Olgiati, S. , De Rosa, A. , Quadri, M. , Criscuolo, C. , Breedveld, G. J. , Picillo, M. , … Bonifati, V. (2014). PARK20 caused by SYNJ1 homozygous Arg258Gln mutation in a new Italian family. Neurogenetics, 15(3), 183–188. 24816432 10.1007/s10048-014-0406-0

[mgg3341-bib-0011] Taghavi, S. , Chaouni, R. , Tafakhori, A. , Azcona, L. J. , Firouzabadi, S. G. , Omrani, M. D. , … Habibi, S. A. H. (2017). A clinical and molecular genetic study of 50 families with autosomal recessive parkinsonism revealed known and novel gene mutations. Molecular Neurobiology, https://doi.org/10.1007/s12035-017-0535-1. 10.1007/s12035-017-0535-1 PMC5683945 28502045

